# Association of maternal height with pregnancy complications: Analyses of two cohorts

**DOI:** 10.1002/pmf2.70474

**Published:** 2026-10-06

**Authors:** Ruchi Tiwari, Pandora L. Wander, Kwun C. G. Chan, Eeeseung Byun, Daniel A. Enquobahrie

**Affiliations:** ^1^ Department of Epidemiology University of Washington Seattle Washington USA; ^2^ VA Puget Sound Health Care System Seattle Washington USA; ^3^ Department of Medicine University of Washington Seattle Washington USA; ^4^ Department of Biostatistics University of Washington Seattle Washington USA; ^5^ Department of Health Systems and Population Health School of Public Health University of Washington Seattle Washington USA; ^6^ Department of Biobehavioral Nursing and Health Informatics University of Washington Seattle Washington USA

**Keywords:** gestational diabetes, maternal height, preeclampsia

## Abstract

**Background:**

Height is inversely associated with risk of cardiovascular and metabolic conditions in nonpregnant populations. Whether maternal height is independently associated with pregnancy complications, after accounting for prepregnancy weight, remains less clear.

**Objective:**

To investigate associations of maternal height with pregnancy complications, independent of prepregnancy weight, and to examine the role of infant sex and race/ethnicity in these associations.

**Methods:**

We conducted a secondary analysis of data from the Omega and Hyperglycemia and Adverse Pregnancy Outcome (HAPO) prospective cohort studies of pregnant women (total *N* = 8763). Cohort‐specific short (<20th percentile), average (20th–80th percentile), and tall (≥80th percentile) height categories were defined. Gestational diabetes mellitus (GDM), preterm birth (PTB; Omega only), and preeclampsia were considered outcomes. We fit cohort‐specific unadjusted and adjusted modified Poisson regression models with robust standard errors and estimated relative risks (RRs) and 95% confidence intervals (CIs) associated with each 5‐cm greater maternal height. We fit similar models to examine risks among women with short or tall height compared with average height. We evaluated race/ethnicity and infant sex as potential modifiers of height–GDM associations using interaction models.

**Results:**

Each 5‐cm greater maternal height was associated with lower GDM risk in both cohorts, with a modest association in Omega and a larger association in HAPO (Omega: adjusted RR [aRR], 0.99; 95% CI, 0.98, 0.99; HAPO: aRR, 0.84; 95% CI, 0.78, 0.90). Short women had higher GDM risk (Omega: aRR, 1.05; 95% CI, 1.03, 1.07; HAPO: aRR, 1.44; 95% CI, 1.18, 1.75), and tall women had lower GDM risk (Omega: aRR, 0.98; 95% CI, 0.96, 0.99; HAPO: aRR, 0.71; 95% CI, 0.56, 0.89), compared with women with average height. In Omega, linear height was associated with slightly lower preeclampsia risk (aRR, 0.99; 95% CI, 0.99, 0.99) and slightly greater PTB risk (aRR, 1.01; 95% CI, 1.00, 1.01). Infant sex or race/ethnicity did not meaningfully modify the association between height and GDM in either cohort.

**Conclusion:**

Maternal height was inversely associated with GDM risk in both cohorts. We also observed small, cohort‐specific associations of height with PTB and preeclampsia in Omega. These findings suggest that maternal height may provide modest information beyond conventional maternal body‐size measures, although its clinical utility for individual risk prediction or targeted monitoring may be limited and requires further evaluation.

## 1 INTRODUCTION

Gestational diabetes mellitus (GDM), preeclampsia, and preterm birth (PTB) are common and clinically important pregnancy complications, affecting approximately 8%–24%, 2.7%–8.2%, and 9%–12% of pregnancies, respectively [1, 2, 3]. These pregnancy complications have been consistently associated with cardiometabolic risk factors such as hypertension and later‐life morbidity and mortality from cardiovascular and metabolic diseases among mothers and their infants [4, 5]. Older age, family history, physical activity, and obesity, measured by body mass index (BMI), are well‐established risk factors for these complications, whereas maternal height remains less clearly understood as an independent marker of pregnancy risk [6, 7, 8].

Increasing evidence suggests an inverse association of height with the risk of cardiovascular and metabolic conditions among men and nonpregnant women [9, 10, 11, 12, 13, 14]. Studies of height and pregnancy complications, however, have reported mixed findings despite a substantial existing literature [15, 16, 17, 18, 19, 20, 21]. Prior evidence, including the systematic review by Han et al. and large population‐based studies, has linked shorter maternal height with adverse birth outcomes such as PTB and low birthweight [22]. More recent work has also examined maternal height in relation to hypertensive disorders of pregnancy, including preeclampsia [23]. Several studies have reported associations of short height with a greater risk of GDM [16, 17, 21, 24], preeclampsia [18, 19, 20, 22, 23, 25], and PTB [21, 26]. Findings from other observational studies, however, did not support these associations [18, 27]. Further, the independent association of height, after accounting for other maternal body‐size measures, particularly maternal prepregnancy weight (ppW), has not been consistently evaluated across multiple pregnancy outcomes [19, 20, 25, 28]. Despite recent evidence of sex‐specific associations of maternal exposures with pregnancy outcomes [29, 30] and race/ethnicity‐specific associations of height with GDM [31], research on whether height–GDM associations differ by infant sex or maternal race/ethnicity remains limited.

Inconsistencies in previous findings may be related to differences in study design, outcome definitions, population composition, exposure ascertainment, and adjustment for important confounders and precision variables, particularly ppW. These differences may also reflect limited generalizability from racially/ethnically homogeneous populations, lack of consideration of potential effect modifiers such as infant sex and race/ethnicity, and potential misclassification of exposure due to self‐reported height.

The objective of the current study was to investigate associations of maternal height, independent of ppW, with pregnancy complications (GDM, preeclampsia, and PTB) and examine whether these associations persisted after adjustment for other established risk factors. By evaluating multiple pregnancy complications across two cohorts using a consistent analytic framework, this study contributes evidence on the reproducibility, comparative magnitude, and generalizability of these associations beyond conventional maternal body‐size measures. We further examined whether infant sex or maternal race/ethnicity modified the associations between height and GDM.

## 2 METHODS

### 2.1 Study settings and study populations

Data from the Omega and the Hyperglycemia and Adverse Pregnancy Outcome (HAPO) cohorts were used in this study. Details on the study objectives, study designs, inclusion and exclusion criteria, and data collection have been reported in prior publications [32, 33, 34, 35]. Briefly, Omega study participants were recruited from among pregnant women attending prenatal care clinics affiliated with two major hospitals between 1996 and 2008. Out of 5063 eligible women, 4602 (91%) provided informed consent and were enrolled in the cohort.

Study participants in the HAPO study were recruited among pregnant women less than 31 weeks of gestation from 15 field centers located in Asia, Australia, Europe, North America, and the Middle East, between July 2000 and April 2006. If glucose measurements were made outside of the HAPO study after initial enrollment, or if the blood glucose level exceeded set thresholds (fasting: >105 mg/dL, or 2 h: >200 mg/dL, or random: >160 mg/dL, or any glucose level <45 mg/dL), the participant was excluded. All participants provided written informed consent. Of 53,295 eligible women (all centers), 28,562 (53.59%) agreed to participate and among those, 25 505 (89.29%) completed an oral glucose tolerance test (OGTT). For the HAPO study, we had access to publicly available data on 4994 genotyped mother/infant pairs which we downloaded from the Database of Genotypes and Phenotypes (dbGaP) website, under phs000096.v4.p1.

The current study involved secondary analysis using de‐identified data; hence, IRB review and approval were not required. We excluded participants with pre‐existing diabetes or hypertension, multiple pregnancy, or those with missing information on height, GDM, preeclampsia, or PTB, including extreme height (height <140 cm or >200 cm). Our final analytic sample included 3880 women from the Omega study and 4843 from the HAPO study.

### 2.2 Data collection

In Omega, information on maternal sociodemographic and medical characteristics, family and medical history, height, ppW, smoking, and alcohol drinking habits was collected through interviewer‐administered questionnaire at an average of 15 weeks of gestation. Information on gestational age at delivery, maternal health during the pregnancy and at the time of delivery, and pregnancy outcomes was abstracted from hospital labor and delivery medical records and clinic records by trained study personnel after delivery [16].

The participants in HAPO underwent 75 g OGTT as close to 28 weeks’ gestation as possible. At the OGTT visit, height, weight, and blood pressure were measured while other prenatal information, including sociodemographic characteristics and participant's report of ppW, was collected using questionnaires. Following OGTT, routine pregnancy care was continued at the field centers.

For each study population, we defined cohort‐specific quintiles of height (centimeter) using self‐reported height in Omega and measured height in HAPO. Similar to a previous study [21], we categorized height as short height (<20th percentile), average height (20th–80th percentile), and tall height (>80th percentile). These cohort‐specific categories were intended to compare relative stature within each cohort; continuous height models were considered the primary analysis for cross‐cohort interpretation.

Diagnosis of GDM was made post hoc in HAPO data based on current International Association of Diabetes and Pregnancy Study Groups (IADPSG) criteria [36]. Using blood glucose values from OGTT performed in the fasting state, GDM was diagnosed if any of the following cut‐offs were met: (1) fasting ≥92 mg/dL (≥5.2 mmol/L), (2) 1 h ≥180 mg/dL (≥10 mmol/L), or (3) 2 h ≥ 153 mg/dL (≥8.5 mmol/L). In Omega, diagnosis of GDM was based on American Diabetes Association guidelines in use at the time [36, 37]. All women were screened for GDM between 24 and 28 weeks' gestation using a 50‐g glucose challenge test and a subsequent 3‐h OGTT with a 100‐g glucose load if the post‐load glucose was ≥140 mg/dL. Women were diagnosed with GDM if ≥ 2 of the following cut‐offs were met: (1) fasting ≥105 mg/dL (≥ 5.8 mmol/L), (2) 1 h ≥ 190 mg/dL (≥10.6 mmol/L), (3) 2 h ≥165 mg/dL (≥ 9.2 mmol/L), or (4) 3 h ≥145 mg/dL (≥ 8.1 mmol/dL), and no indication of prior chronic diabetes was noted. Preeclampsia diagnosis was based on clinical records in both cohorts [36]. PTB was defined as birth before 37 completed weeks of gestation estimated using the date of the last menstrual period (LMP) or early pregnancy ultrasound in both cohorts. An ultrasound was performed between 6 and 24 weeks' gestation [38] in HAPO if the participant was not certain of her LMP.

### 2.3 Statistical analysis

We summarized selected characteristics of participants overall and by quintiles of height and additionally compared our analytic cohort with the full HAPO cohort, using mean and standard deviation (SD) for continuous variables and number (count) and percent of total for categorical variables. We also summarized absolute event rates for each outcome to aid interpretation of the clinical magnitude of associations. To estimate height‐outcome associations, we fit cohort‐specific unadjusted and adjusted modified Poisson regression models with robust standard errors [39] and estimated relative risks (RRs) and 95% confidence intervals (CIs) associated with (1) each 5‐cm greater height (linear) and (2) tall height and short height, compared with cohort‐specific average height (categorical).

To estimate adjusted associations between height and the outcomes, the following covariates were identified a priori and added to the unadjusted regression models, either as confounders or precision variables: maternal age (years), self‐reported ppW (kg), and gestational age at delivery (weeks) were included as continuous variables. Gestational age at delivery was included as a precision variable; because it may be affected by preeclampsia and defines PTB, estimates for these outcomes were interpreted as descriptive associations rather than causal effects. Post–high school education, married marital status (Omega only), nulliparity, smoking during pregnancy, alcohol use during pregnancy, and infant male sex were included as binary variables with yes/no categories. Study sites were included as a grouped categorical variable (HAPO only), and maternal race/ethnicity was categorized as White, Black, Asian, or Hispanic in adjusted models in both studies.

To examine potential effect modification of the height–GDM association, we included interaction terms between height and each effect modifier (infant sex: male/female and race/ethnicity: categorized as above) in the fully adjusted regression models for GDM. We evaluated multiplicative interaction using Wald tests for the interaction terms and report interaction *p* values to describe evidence of heterogeneity. Regression models were fit among strata defined by effect modifiers when interaction estimates suggested heterogeneity in the height–GDM association.

In HAPO, we conducted sensitivity analyses using fasting, 1‐h, and 2‐h OGTT values to identify linear associations between height and blood glucose levels (instead of GDM). We emphasized effect estimates and 95% CIs as the primary metrics for interpretation; *p* values are reported for interaction tests to describe evidence of heterogeneity. All calculations and analyses were done separately for each of the two cohorts and for each outcome. Analyses were conducted using R Studio software (R Version 4.1.1 [2021‐08‐10]).

## 3 RESULTS

Mean height for women in the analytic population was 168 cm (SD: 7.68) in Omega and 161 cm (SD: 7.51) in HAPO. We calculated the cut‐offs for short, average, and tall heights (Tables S1 and S2). Overall, 85.3% of participants in Omega and 30.0% of participants in HAPO were White, while 61.7% and 67.9%, respectively, were nulliparous (Table 1). Prepregnancy weight was available for all Omega participants included in the analytic sample and was missing for 571 HAPO participants (11.8%). The prevalence of GDM, preeclampsia, and PTB in Omega was 4.9%, 1.8%, and 7.8%, respectively, while the corresponding values in HAPO were 15.0%, 2.1%, and 0.1%, respectively.

**TABLE 1:** Selected characteristics of pregnant women enrolled in the Omega study and HAPO study.

Characteristics^a^	Omega (*N* = 3880)	HAPO (*N* = 4843)
**Mother**		
Race/ethnicity		
White	3309 (85.3%)	1451 (30.0%)
Black	66 (1.7%)	1291 (26.7%)
Asian	302 (7.8%)	871 (18.0%)
Hispanic	203 (5.2%)	1230 (25.4%)
Age (years)	32.7 (4.48)	28.4 (5.87)
Age ≥35 years	1343 (34.6%)	704 (14.5%)
Married	3529 (91.0%)	–
Nulliparous^b^	2394 (61.7%)	3288 (67.9%)
Post–high school education^c^	3525 (90.9%)	2367 (48.9%)
Smoked during pregnancy^d^	770 (19.8%)	219 (4.5%)
Intake of any alcohol during pregnancy^e^	362 (9.3%)	349 (7.2%)
Prepregnancy weight (kg)^f^	65.8 (13.5)	64.8 (13.5)
Prepregnancy body mass index (kg/m^2^)^f^	23.4 (4.51)	25.2 (4.91)
Prepregnancy body mass index category (kg/m^2^)		
Underweight (<18.5)	170 (4.4%)	181 (3.7%)
Normal (18.5–24.9)	2775 (71.5%)	2177 (45.0%)
Overweight (25–29.9)	641 (16.5%)	1326 (27.4%)
Obesity (≥30)	294 (7.6%)	588 (12.1%)
Prepregnancy body mass index category (kg/m^2^) using Asia‐Pacific criteria		
Underweight (<18.5)	170 (4.4%)	181 (3.7%)
Normal (18.5–22.9)	2111 (54.4%)	1329 (27.4%)
Overweight (23–24.9)	664 (17.1%)	848 (17.5%)
Obesity (≥25)	935 (24.1%)	1914 (39.5%)
Gestational age at delivery (weeks)^g^	38.9 (2.07)	39.7 (1.23)
Gestational diabetes^h^	192 (4.9%)	725 (15.0%)
Preeclampsia	70 (1.8%)	101 (2.1%)
**Newborn**		
Male infant^i^	1988 (51.2%)	2445 (50.5%)
Preterm (<37 weeks)	301 (7.8%)	6 (0.1%)
Birth weight (g)	3460 (599)	3290 (470)

^a^ Values are *N* (%) or mean (standard deviation).

^b^ Information on parity missing for one subject in Omega and 588 subjects in HAPO.

^c^ Educational status missing for 231 subjects in Omega and 239 subjects in HAPO.

^d^ Smoking status missing for 247 subjects in Omega.

^e^ Alcohol intake missing for 54 subjects in Omega.

^f^ Prepregnancy weight and BMI were complete for Omega and missing for 571 subjects in HAPO (11.8%).

^g^ Gestational age at delivery missing for four subjects in Omega.

^h^ Diagnosis of gestational diabetes was made post hoc based on IADPSG criteria (one or more values exceeding the thresholds: fasting ≥92 mg/dL, 1 h ≥180 mg/dL, and 2 h ≥153 mg/dL).

^i^ Infant sex missing for 11 subjects in Omega.

Figure 1 shows the unadjusted and adjusted RR (aRR) obtained from modified Poisson regression models for the outcomes in relation to maternal height. Due to the limited number of preterm delivery cases (*N* = 6), we did not conduct any analysis for PTB in HAPO. After covariate adjustment, each 5‐cm greater maternal height was associated with lower GDM risk in both cohorts, with a modest association in Omega and a larger association in HAPO (Omega: aRR, 0.99; 95% CI, 0.98, 0.99; HAPO: aRR, 0.84; 95% CI, 0.78, 0.90). Women with short height, compared with women with average height, had higher GDM risk in both studies (Omega: aRR, 1.05; 95% CI, 1.03, 1.07; HAPO: aRR, 1.44; 95% CI, 1.18, 1.75). Tall women had lower GDM risk compared with women with average height (Omega: aRR, 0.98; 95% CI, 0.96, 0.99; HAPO: aRR, 0.71; 95% CI, 0.56, 0.89). In Omega, linear height was associated with a slightly lower risk of preeclampsia (aRR, 0.99; 95% CI, 0.99, 0.99) and with a slightly higher risk of PTB (aRR, 1.01; 95% CI, 1.00, 1.01). We did not observe a clear association of height with preeclampsia in HAPO (aRR, 0.89; 95% CI, 0.75, 1.08).

**FIGURE 1:**
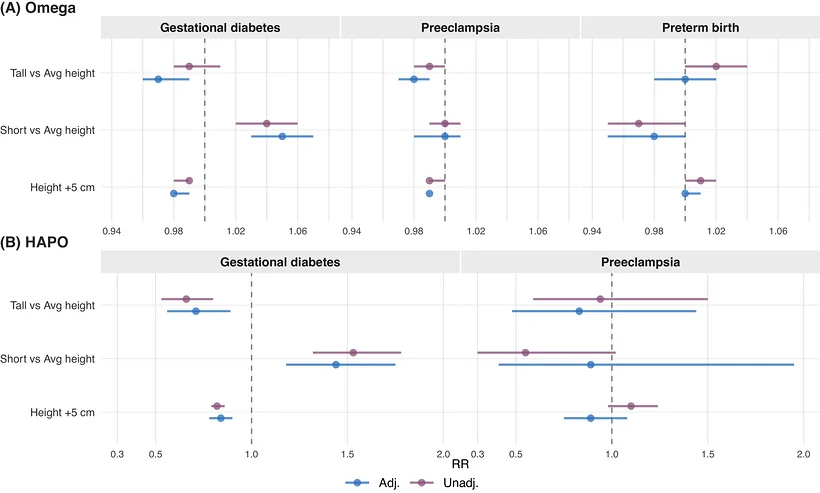
Associations of maternal height with gestational diabetes, preeclampsia, and preterm birth among Omega and HAPO study participants. Unadj (unadjusted) model included terms for linear height or categorical height (indicator variable; short height, tall height, average height) and each outcome (binary). Adj (adjusted) models adjust for continuous variables—maternal age (years), prepregnancy weight (kg), gestational age (weeks)—and binary variables—post–high school education, nulliparity, smoking during pregnancy, alcohol use during pregnancy, and male infant. Omega study additionally adjusts for marital status (binary) and HAPO study additionally adjusts for study site (grouped categorical variable for six study sites). Gestational age at delivery was included as a precision variable; estimates for preeclampsia and PTB should be interpreted descriptively rather than causally. Relative risk (RR) for linear height represents the relative risk of outcome associated with 5‐cm greater maternal height. Wald *p* values are shown for reference; interpretation emphasizes effect estimates and 95% CIs. Definitions of short and tall heights were cohort specific. *p* values for categorical height represent Wald *p* values for terms β_1_ and β2_2_ from the model, log (E(Y|X_height_, X_covariates_)) = β_0_+ β_1_× ln(X_height‐short_) + β_2_× ln(X_height‐tall_) + β × Covariates, where Y is the outcome, β_1_= 0 tests whether the risk of outcome comparing women with short height to women with average height is different from 1, and beta β_2_= 0 tests whether the risk of outcome comparing women with tall height to women with average height is different from 1.

We did not observe evidence that infant sex or race/ethnicity meaningfully modified the height–GDM association in either cohort. Interaction *p* values for infant sex were 0.90 in Omega and 0.30 in HAPO; interaction *p* values for race/ethnicity were >0.90 in Omega and 0.50 in HAPO (Table 2). Effect modifier‐stratified models did not show meaningful differences in associations across categories defined by effect modifiers. Findings from sensitivity analyses showed inverse associations of maternal height with blood glucose levels in HAPO, similar to our main findings reported above (Table 3).

**TABLE 2:** The associations of short and tall maternal heights with gestational diabetes by maternal race/ethnicity and infant sex among Omega and HAPO study participants.

	Omega study^a^			HAPO study^a^		
	Short height	Tall height		Short height	Tall height	
	RR (95% CI)	RR (95% CI)	*p* value^b^	RR (95% CI)	RR (95% CI)	*p* value^b^
Infant sex			0.90			0.30
Male infant	1.07 (1.04, 1.10)	0.99 (0.97, 1.01)		1.04 (0.98, 1.12)	0.95 (0.91, 0.98)	
Female infant	1.03 (1.00, 1.06)	0.97 (0.95, 0.99)		1.10 (1.04, 1.16)	0.97 (0.94, 1.01)	
Race/ethnicity			>0.90			0.50
White	1.05 (1.03, 1.07)	0.98 (0.96, 0.99)		1.10 (1.00, 1.22)	0.96 (0.92, 0.99)	
Black	1.02 (0.87, 1.19)	0.94 (0.78, 1.14)		0.99 (0.94, 1.05)	0.99 (0.95, 1.02)	
Asian	1.05 (0.97, 1.14)	0.97 (0.82, 1.15)		1.09 (1.02, 1.16)	1.19 (0.80, 1.76)	
Hispanic	1.07 (0.98, 1.18)	1.02 (0.93, 1.12)		1.08 (1.00, 1.17)	0.90 (0.84, 0.98)	

Abbreviations: CI, confidence interval; RR, relative risk.

^a^ The estimates are in comparison to average‐height women.

^b^ *p* value denotes the *p* value of the interaction term between height and effect modifier in the adjusted model.

**TABLE 3:** Associations of maternal height with fasting, 1‐h, and 2‐h blood glucose levels among HAPO study participants.

	Fasting blood glucose^[Link]^, ^[Link]^		1‐h blood glucose^[Link]^, ^[Link]^		2‐h blood glucose^[Link]^, ^[Link]^	
	aβ (95% CI)	*p* value	aβ (95% CI)	*p* value	aβ (95% CI)	*p* value
Linear height (+5 cm)^c^	−0.54 (−0.73, −0.35)	<0.001	−3.08 (−3.91, −2.26)	<0.001	−2.96 (−3.58, −2.33)	<0.001
Categorical height^d^						
Short	0.97 (0.29, 1.65)	0.01	5.83 (2.89, 8.76)	<0.001	4.73 (2.51, 6.94)	<0.001
Tall	−1.25 (−1.82, −0.67)	0.00	−5.25 (−7.73, −2.77)	<0.001	−6.14 (−8.01, −4.27)	<0.001

^a^Model included term (s) for height, measured in cm, and blood glucose level, measured in mg/dL.

^b^Models adjust for continuous variables—maternal age (years), prepregnancy weight (kg), gestational age (weeks)—and binary variables—post–high school education, nulliparity, smoking during pregnancy, alcohol use during pregnancy, male infant, and study site (grouped categorical variable for six study sites).

^c^ß for linear height represents the change in blood glucose level in mg/dL per 5 cm greater height of the mother.

Wald P‐values are shown for reference; interpretation emphasizes effect estimates and 95% CIs.

^d^Definitions of short and tall heights were cohort specific. *p* values for categorical height represent Wald *p* values.

## 4 DISCUSSION

In the current study, greater maternal height was associated with lower GDM risk, independent of ppW, across both Omega and HAPO cohorts. These associations were not meaningfully modified by infant sex or maternal race/ethnicity. We also observed small associations of maternal height with PTB and preeclampsia in Omega, although these findings should be interpreted cautiously given their limited magnitude and cohort‐specific nature. Although these small effect sizes may have limited utility for individual‐level prediction, they may still have clinical or public‐health relevance when outcomes have severe consequences or when modest shifts in risk affect large populations.

Our findings of inverse height–GDM associations are generally consistent with previous reports in the literature [16, 17, 21, 24]. A USA‐based analysis that included 135,861 pregnancies found that with every 5‐cm greater self‐reported or measured height, the risk of GDM was reduced by 20% (OR, 0.80; 95% CI, 0.78, 0.82) [17]. A previous analysis of the Omega cohort (*N* = 1644) reported lower odds of GDM among taller mothers (aOR, 0.40; 95% CI, 0.17, 0.95, comparing fourth quartile [>170 cm] vs first quartile [<160 cm]. [16] Our analysis extends this prior Omega finding by evaluating height using both linear and cohort‐specific categorical definitions, by accounting for ppW, and by examining consistency in an independent cohort.

Among all California births that occurred between 2007 and 2010 (*N* = 2,094,220), a previous study that examined the associations of height with GDM, preeclampsia, and PTB [21] reported higher odds of GDM (aOR, 1.20; 95% CI, 1.19, 1.22), preeclampsia (aOR, 1.04; 95% CI, 1.02, 1.06), and PTB (aOR, 1.11; 95% CI, 1.10, 1.13), comparing women with short height to women with average height. Similarly, tall height, compared with average height, was associated with lower odds of GDM (aOR, 0.81; 95% CI, 0.80, 0.82) and PTB (aOR, 0.91; 95% CI, 0.90, 0.92), and slightly higher odds of preeclampsia (aOR, 1.03; 95% CI, 1.01, 1.05). Taken together with prior evidence, our findings support an inverse association between maternal height and GDM, with a consistent direction of association across both Omega and HAPO. In contrast, associations with preeclampsia and PTB were small and less consistent with previous reports, suggesting that these relationships may be more context‐dependent and should be interpreted with careful consideration of effect size, precision, outcome prevalence, and cohort characteristics. The observation of a similar direction of association in two cohorts that differed in racial/ethnic composition, body‐size distribution, height ascertainment, and GDM diagnostic criteria supports the generalizability of the overall height–GDM relationship, although differences in effect magnitude warrant cohort‐specific interpretation.

Unlike the California‐based and other studies [19, 20, 23, 25, 28], we did not observe clear associations of height with preeclampsia in HAPO, but we observed small associations of height with preeclampsia and PTB in Omega. A positive association between height and preeclampsia in Californian pregnancies was reported, in contrast to what we found in Omega. However, for a subset of normal‐weight women, the magnitude, direction, and precision of association were similar to our findings from Omega (aOR, 0.95; 95% CI, 0.92, 0.98), suggesting that differences in population composition and maternal body size may partly explain the discrepant findings. A study conducted in Uruguay [27] did not observe an association between height and the risk of preeclampsia.

Despite the similarities, certain factors may account for the different findings in Omega, HAPO, and other previous studies. We adjusted for ppW, but previous studies did not consistently evaluate height independent of ppW [19, 20, 25, 28]. The study populations also varied substantially. Omega included predominantly White women, whereas HAPO included a geographically and racially/ethnically heterogeneous group of women. In Omega, mean height ranged from 162 cm among Asian women to 168 cm among White women, whereas in HAPO, the range was 154 cm among Asian women to 164 cm among White and Black women. Most women in Omega were normal weight (71.5%), while 16.5% were overweight based on standard BMI cut‐offs. In comparison, less than half of women in HAPO were normal weight (45.0%), and 27.4% were overweight. The diagnostic thresholds for glucose values used in the definition of GDM in HAPO also differed from those used in Omega. Differences in height ascertainment, self‐reported in Omega and measured in HAPO, may also have contributed to heterogeneity in effect estimates. Cohort‐specific height categories supported within‐cohort interpretation but limit direct clinical applicability and comparability across cohorts because the absolute height thresholds differed between Omega and HAPO. Notably, given the small number of preeclampsia and PTB cases in each height category, we may have been underpowered to detect associations of height with these outcomes. The prevalence of PTB in our HAPO analytic sample was lower than the prevalence in the larger HAPO cohort (*N* = 25,505) (0.1% vs. 6.9%), but we were unable to determine whether this difference was due to additional inclusion criteria for genotyping the study participants. Together, these findings suggest that the association between maternal height and pregnancy complications may vary by study population, analytic approach, and outcome characterization. While our study is one of the few to investigate potential roles of maternal race/ethnicity or infant sex in height‐GDM associations, we did not find evidence that these factors meaningfully modified the observed associations.

Several biological mechanisms provide plausibility for the associations that we observed. Shared genetic factors may contribute to both short stature and adverse pregnancy outcomes. Epigenetic adaptation during fetal life in response to nutritional insufficiency may contribute to later preeclampsia and GDM risk during pregnancy [40, 41]. Inadequate intrauterine and childhood nutrition may lead to decreased growth hormone activity, resulting in shorter adult height and impaired glucose tolerance in adulthood [42]. However, adult height also reflects early‐life nutrition, socioeconomic conditions, and genetic influences that may not be fully captured in our covariates; residual confounding by these factors may partly explain the observed associations. The association of height with GDM risk could also be partly influenced by glucose testing protocols, because individuals with different body size and body composition are given the same glucose load [21, 43].

Strengths of this study include the use of two well‐characterized prospective cohorts with complementary population characteristics, allowing comparison across distinct study settings and strengthening the generalizability of directionally consistent findings, particularly for height and GDM. There are several limitations of our study that need to be acknowledged. Prepregnancy weight was missing for 571 HAPO participants (11.8%), which limits the strength of conclusions about the independence of maternal height from ppW in this cohort. We did not impute ppW because the available public‐use/genotyped HAPO subset did not provide sufficient information to confidently model the missingness mechanism; instead, we used complete‐case models and sensitivity analyses excluding ppW. Excluding ppW from regression models did not materially change our findings, but residual bias from missing ppW remains possible. Self‐reported height in Omega could have been overestimated [43, 44], and some misclassification due to overreporting or underreporting of reported measures, such as ppW, is possible. The observed difference in effect sizes may partly reflect the use of reported height in Omega and measured height in HAPO. As in all observational studies, we could not rule out bias due to residual or unmeasured confounding and cannot infer causality between height and the outcomes. There could be differences between those who agreed to participate in HAPO (53.59%) and those who did not (46.10%) affecting the risk estimates, but we were unable to determine this based on our data. Selection bias because of the inclusion of predominantly term births in the publicly available HAPO data used in our analysis is possible, and valid selection weights could not be constructed from the available analytic data. Similar characteristics of participants in our analytic cohort and the full HAPO cohort provided reassurance that restricting our analysis to term births is unlikely to substantially bias our estimates [45] (Table S3).

In conclusion, we found an inverse association between maternal height and GDM risk in Omega and HAPO. We observed small associations of maternal height with preeclampsia and PTB risk in Omega. Our results suggest that maternal height may provide modest additional information beyond conventional body‐size measures, although its clinical value for risk prediction or targeted monitoring requires further evaluation. Future studies should replicate these findings in larger and more diverse cohorts and use causal‐inference frameworks to clarify whether maternal height is a marker, mediator, or proxy for developmental and cardiometabolic pathways related to pregnancy complications.

## CONFLICT OF INTEREST STATEMENT

The authors declare no conflicts of interest.

## ETHICAL STATEMENTS

Omega study was approved by the institutional review boards of Swedish Medical Center and Tacoma General Hospital. The HAPO study was approved by the institutional review boards at all 15 field centers.

## Supporting information



Supporting information

Supporting information

Supporting information
