# Supplementary material for: Association of maternal height with pregnancy complications: Analyses of two cohorts
Source: Pregnancy (Hoboken). 2026 Oct 6;2(6):e70474. doi: 10.1002/pmf2.70474 (PMC13640055; doi:10.1002/pmf2.70474)
Supplement: Supplementary file 1 [file PMF2-2-e70474-s001.docx]

**S1 Table. Selected characteristics of Omega study participants enrolled between1996 and 2008 by height quintiles**

| Characteristics ^a^ | First quintile^b^ (N = 776) | Second quintile^c^ (N=775) | Third quintile^d^ (N=777) | Fourth quintile^e^ (N=776) | Last quintile^f^ (N=776) | Overall (N=3880) |
| --- | --- | --- | --- | --- | --- | --- |
| Mother |  |  |  |  |  |  |
| Race/ethnicity |  |  |  |  |  |  |
| White | 536 (69.1%) | 659 (845.0%) | 685 (88.2%) | 699 (90.1%) | 730 (94.1%) | 3309 (85.3%) |
| Black | 20 (2.6%) | 14 (1.8%) | 9 (1.2%) | 14 (1.8%) | 9 (1.2%) | 66 (1.7%) |
| Asian | 156 (20.1%) | 64 (8.3%) | 44 (5.7%) | 24 (3.1%) | 14 (1.8%) | 302 (7.8%) |
| Hispanic | 64 (8.2%) | 38 (4.9%) | 39 (5.0%) | 39 (5.0%) | 23 (3.0%) | 203 (5.2%) |
| Age (years) | 32.0 (4.82) | 32.6 (4.53) | 33.0 (4.31) | 33.0 (4.33) | 32.9 (4.32) | 32.7 (4.48) |
| Age ≥ 35 years | 240 (30.9%) | 261 (33.7%) | 284 (36.6%) | 279 (36.0%) | 279 (36.0%) | 1343 (34.6%) |
| Not married | 89 (11.5%) | 65 (8.4%) | 64 (8.2%) | 63 (8.1%) | 70 (9.0%) | 351 (9.0%) |
| Nulliparous ^g^ | 464 (59.8%) | 470 (60.6%) | 497 (64.0%) | 481 (62.0%) | 482 (62.1%) | 2394 (61.7%) |
| Post high school education ^h^ | 662 (85.3%) | 690 (89.0%) | 720 (92.7%) | 722 (93.0%) | 731 (94.2%) | 3525 (90.9%) |
| Smoked during pregnancy ^i^ | 117 (15.1%) | 166 (21.4%) | 155 (19.9%) | 145 (18.7%) | 187 (24.1%) | 770 (19.8%) |
| Intake of any alcohol during pregnancy ^j^ | 69 (8.9%) | 67 (8.6%) | 82 (10.6%) | 78 (10.1%) | 66 (8.5%) | 362 (9.3%) |
| Pre-pregnancy weight (kg) | 59.4 (11.9) | 63.5 (12.5) | 65.1 (11.9) | 68.0 (13.1) | 73.1 (14.2) | 65.8 (13.5) |
| Pre-pregnancy body mass index (kg/m^2^) | 24.0 (4.81) | 23.7 (4.70) | 23.2 (4.24) | 23.1 (4.45) | 22.9 (4.22) | 23.4 (4.51) |
| Pre-pregnancy body mass index category (kg/m^2^) |  |  |  |  |  |  |
| Underweight (<18.5) | 27 (3.5%) | 30 (3.9%) | 31 (4.0%) | 39 (5.0%) | 43 (5.5%) | 170 (4.4%) |
| Normal (18.5–24.9) | 542 (69.8%) | 553 (71.4%) | 560 (72.1%) | 560 (72.2%) | 560 (72.2%) | 2775 (71.5%) |
| Overweight (25–29.9) | 122 (15.7%) | 126 (16.3%) | 131 (16.9%) | 125 (16.1%) | 137 (17.7%) | 641 (16.5%) |
| Obesity (≥ 30) | 85 (11.0%) | 66 (8.5%) | 55 (7.1%) | 52 (6.7%) | 36 (4.6%) | 294 (7.6%) |
| Gestational age at delivery (weeks) ^k^ | 38.8 (2.71) | 38.8 (2.00) | 39.0 (1.55) | 39.0 (1.93) | 39.0 (1.97) | 38.9 (2.07) |
| Gestational diabetes | 67 (8.6%) | 36 (4.6%) | 30 (3.9%) | 33 (4.3%) | 26 (3.4%) | 192 (4.9%) |
| Pre-eclampsia | 13 (1.7%) | 22 (2.8%) | 10 (1.3%) | 15 (1.9%) | 10 (1.3%) | 70 (1.8%) |
| Newborn |  |  |  |  |  |  |
| Male infant ^l^ | 414 (53.4%) | 390 (50.3%) | 378 (48.6%) | 406 (52.3%) | 400 (51.5%) | 1988 (51.2%) |
| Preterm (<37 weeks) | 79 (10.2%) | 77 (9.9%) | 50 (6.4%) | 52 (6.7%) | 43 (5.5%) | 301 (7.8%) |
| Birth weight | 3300 (546) | 3390 (567) | 3480 (613) | 3520 (491) | 3620 (708) | 3460 (599) |

^a^ Values are N (%) or mean (Standard Deviation)

^b^ First quintile, height <161.35 cm.

^c^ Second quintile, height ≥ 161.35 cm to <165.44 cm.

^d^ Third quintile, height ≥ 165.44 cm to <169.53 cm.

^e^ Fourth quintile, height ≥ 169.53 cm to >173.99 cm.

^f^ Fifth quintile, height ≥ 173.99 cm

^g^ Information on parity missing for 1 subject. For each quintile: 1 (quintile 1) was missing.

^h^ Educational status missing for 231 subjects. For each quintile: 82 (quintile 1), 58 (quintile 2), 33 (quintile 3), 38 (quintile 4), 20 (quintile 5)

^i^ Smoking status missing for 247 subjects. For each quintile: 85 (quintile 1), 62 (quintile 2), 37 (quintile 3), 41 (quintile 4), 22 (quintile 5)

^j^ Alcohol intake missing for 54 subjects. For each quintile: 13 (quintile 1), 14 (quintile 2), 11 (quintile 3), 3 (quintile 4), 13 (quintile 5)

^k^ Gestational age at delivery missing for 4 subjects. For each quintile: 2 (quintile 3), 2 (quintile 4)

^l^ Infant sex missing for 11 subjects. For each quintile: 1 (quintile 1), 2 (quintile 3), 1 (quintile 4), 7 (quintile 5)
