# Supplementary material for: Association of maternal height with pregnancy complications: Analyses of two cohorts
Source: Pregnancy (Hoboken). 2026 Oct 6;2(6):e70474. doi: 10.1002/pmf2.70474 (PMC13640055; doi:10.1002/pmf2.70474)
Supplement: Supplementary file 2 [file PMF2-2-e70474-s002.docx]

**S3 Table****. Maternal and infant characteristics of the analytic cohort and the full cohort in HAPO study**

| Characteristics ^ab^ |  | Analytic Cohort (N=4843) |  | Full Cohort (N=23316) | Range of Means among study centers in Full Cohort |
| --- | --- | --- | --- | --- | --- |
| Mother |  |  |  |  |  |
| Age (years) |  | 28.4 (5.87) |  | 29.2 (5.80) | 25.4-33.6 |
| Any smoking during pregnancy |  | 219 (4.5%) |  | 1581 (6.8%) | 0.2-23.6% |
| Any alcohol during pregnancy |  | 349 (7.2%) |  | 1612 (6.9%) | 0.1-26.5% |
| Pre-pregnancy body mass index (kg/m^2^) |  | 25.2 (4.91) |  | 27.7 (5.10) | 24.4-29.9 |
| Gestational age at delivery (weeks) |  | 39.7 (1.23) |  | 39.4 (1.7) | 38.7-39.9 |
| Blood glucose level at OGTT (mg/dl) |  |  |  |  |  |
| Fasting |  | 81.4 (7.26) |  | 80.9 (6.90) | 78.2-83.7 |
| One hour |  | 134 (31.7) |  | 134.1 (30.9) | 119.5-148.2 |
| Two hour |  | 112 (23.5) |  | 111 (23.5) | 99.6-120.9 |
| Pre-eclampsia |  | 101 (2.1%) |  | 1116 (4.8) | 1.4-11.4% |
| Newborn |  |  |  |  |  |
| Male infant |  | 2445 (50.5%) |  | 12003 (51.5%) | 49.3-54.0% |
| Preterm (<37 weeks) |  | 6 (0.1%) |  | 1608 (6.9%) | 3.9-9.1% |
| Birth weight (g) |  | 3290 (470) |  | 3292 (529) | 3109–3526 |

^a^ Values are N (%) or mean (Standard Deviation)

^b^Information on data for full cohort was obtained from Reference (40)
