# Supplementary material for: Association of maternal height with pregnancy complications: Analyses of two cohorts
Source: Pregnancy (Hoboken). 2026 Oct 6;2(6):e70474. doi: 10.1002/pmf2.70474 (PMC13640055; doi:10.1002/pmf2.70474)
Supplement: Supplementary file 3 [file PMF2-2-e70474-s003.docx]

**S2 Table. Selected characteristics of HAPO study participants enrolled at field centers between July 2000 and April 2006 by height quintiles**

| Characteristics ^a^ | First quintile^b^ (N=936) | Second quintile^c^ (N=996) | Third quintile^d^ (N=913) | Fourth quintile^e^ (N=940) | Last quintile^f^ (N=1058) | Overall (N=4843) |
| --- | --- | --- | --- | --- | --- | --- |
| Mother |  |  |  |  |  |  |
| Race/ethnicity |  |  |  |  |  |  |
| White | 74 (7.9%) | 217 (21.8%) | 285 (31.2%) | 376 (40.0%) | 499 (47.2%) | 1451 (30.0%) |
| Black | 91 (9.7%) | 188 (18.9%) | 261 (28.6%) | 301 (32.0%) | 450 (42.5%) | 1291 (26.7%) |
| Hispanic | 152 (16.2%) | 188 (18.9%) | 229 (25.1%) | 206 (21.9%) | 96 (9.1%) | 871 (18.0%) |
| Asian | 619 (66.1%) | 403 (40.5%) | 138 (15.1%) | 57 (6.1%) | 13 (1.2%) | 1230 (25.4%) |
| Age (years) | 28.3 (5.64) | 28.1 (5.68) | 28.4 (5.91) | 28.5 (5.90) | 28.7 (6.19) | 28.4 (5.87) |
| Age ≥ 35 years | 122 (13.0%) | 126 (12.7%) | 129 (14.1%) | 139 (14.8%) | 188 (17.8%) | 704 (14.5%) |
| Nulliparous ^g^ | 502 (53.6%) | 617 (61.9%) | 625 (68.5%) | 703 (74.8%) | 841 (79.5%) | 3288 (67.9%) |
| Post high school education ^h^ | 257 (27.5%) | 438 (44.0%) | 482 (52.8%) | 535 (56.9%) | 655 (61.9%) | 2367 (48.9%) |
| Smoking habits |  |  |  |  |  |  |
| Smoking during pregnancy | 13 (1.4%) | 39 (3.9%) | 47 (5.1%) | 59 (6.3%) | 61 (5.8%) | 219 (4.5%) |
| Number of cigarettes/days during pregnancy | 1.02 (0.163) | 1.05 (0.253) | 1.07 (0.305) | 1.08 (0.319) | 1.07 (0.304) | 1.06 (0.276) |
| Alcohol drinking habits |  |  |  |  |  |  |
| Alcohol during pregnancy | 22 (2.4%) | 55 (5.5%) | 69 (7.6%) | 84 (8.9%) | 119 (11.2%) | 349 (7.2%) |
| Number of alcoholic drinks/days during pregnancy |  |  |  |  |  |  |
| <1 per day | 19 (2.0%) | 55 (5.5%) | 68 (7.4%) | 84 (8.9%) | 116 (11.0%) | 342 (7.1%) |
| 1–2 per day | 1 (0.1%) | 0 (0%) | 0 (0%) | 0 (0%) | 3 (0.3%) | 4 (0.1%) |
| Illicit drug use in pregnancy | 0 (0%) | 3 (0.3%) | 5 (0.5%) | 7 (0.7%) | 8 (0.8%) | 23 (0.5%) |
| Pre-pregnancy weight (kg) ^i^ | 58.7 (9.65) | 62.5 (11.4) | 64.6 (12.7) | 67.4 (14.0) | 70.9 (15.9) | 64.8 (13.5) |
| Pre-pregnancy body mass index (kg/m^2^) ^i^ | 26.1 (4.18) | 25.6 (4.67) | 25.1 (4.96) | 24.9 (5.17) | 24.3 (5.32) | 25.2 (4.91) |
| Pre-pregnancy body mass index category (kg/m^2^) |  |  |  |  |  |  |
| Underweight (<18.5) | 10 (1.1%) | 17 (1.7%) | 35 (3.8%) | 37 (3.9%) | 82 (7.8%) | 181 (3.7%) |
| Normal (18.5–24.9) | 394 (42.1%) | 445 (44.7%) | 415 (45.5%) | 444 (47.2%) | 479 (45.3%) | 2177 (45.0%) |
| Overweight (25–29.9) | 356 (38.0%) | 301 (30.2%) | 250 (27.4%) | 215 (22.9%) | 204 (19.3%) | 1326 (27.4%) |
| Obesity (≥ 30) | 131 (14.0%) | 128 (12.9%) | 107 (11.7%) | 120 (12.8%) | 102 (9.6%) | 588 (12.1%) |
| Gestational age at delivery (weeks) | 39.5 (1.24) | 39.6 (1.23) | 39.7 (1.18) | 39.8 (1.24) | 39.8 (1.22) | 39.7 (1.23) |
| Blood glucose level at OGTT (mg/dl) |  |  |  |  |  |  |
| Fasting | 81.0 (7.38) | 81.1 (6.87) | 81.9 (7.49) | 81.9 (7.41) | 81.4 (7.14) | 81.4 (7.26) |
| One hour | 145 (33.0) | 137 (31.8) | 133 (31.1) | 131 (30.7) | 127 (28.8) | 134 (31.7) |
| Two hour | 119 (25.7) | 115 (23.7) | 110 (22.5) | 110 (22.3) | 106 (21.3) | 112 (23.5) |
| Gestational diabetes ^j^ | 209 (22.3%) | 164 (16.5%) | 129 (14.1%) | 122 (13.0%) | 101 (9.5%) | 725 (15.0%) |
| Pre-eclampsia | 12 (1.3%) | 15 (1.5%) | 27 (3.0%) | 24 (2.6%) | 23 (2.2%) | 101 (2.1%) |
| Newborn |  |  |  |  |  |  |
| Male infant | 463 (49.5%) | 500 (50.2%) | 455 (49.8%) | 477 (50.7%) | 550 (52.0%) | 2445 (50.5%) |
| Preterm (<37 weeks) | 0 (0%) | 0 (0%) | 1 (0.1%) | 2 (0.2%) | 3 (0.3%) | 6 (0.1%) |
| Birth weight (g) | 3110 (408) | 3210 (428) | 3290 (458) | 3370 (465) | 3450 (505) | 3290 (470) |

^a^ Values are N (%) or mean (Standard Deviation)

^b^ First quintile, height <154.05 cm.

^c^ Second quintile, height ≥ 154.05 cm to <158.75 cm.

^d^ Third quintile, height ≥ 158.75 cm to <162.56 cm.

^e^ Fourth quintile, height ≥ 162.56 cm to <167.00 cm.

^f^ Fifth quintile, height ≥ 167.00 cm

^g^ Information on parity missing for 588 subjects. For each quintile: 261 (quintile 1), 173 (quintile 2), 81 (quintile 3), 47 (quintile 4), 26 (quintile 5)

^h^ Educational status missing for 239 subjects. For each quintile: 90 (quintile 1), 44 (quintile 2), 38 (quintile 3), 36 (quintile 4), 31 (quintile 5)

^i^ Pre-pregnancy weight and BMI missing for 571 subjects. For each quintile: 45 (quintile 1), 105 (quintile 2), 106 (quintile 3), 124 (quintile 4), 191 (quintile 5)

^j^ Diagnosis of gestational diabetes was made post hoc based on IADPSG criteria (1 or more values exceeding the thresholds: fasting ≥92 mg/dl, one-hour ≥180 mg/dl, and two-hour ≥153 mg/dl)
